# Prethymic Cytoplasmic CD3 Negative Acute Lymphoblastic Leukemia or Acute Undifferentiated Leukemia: A Case Report

**DOI:** 10.1155/2011/230568

**Published:** 2011-07-28

**Authors:** Elisa Cannizzo, Giovanni Carulli, Luigi Del Vecchio, Antonio Azzarà, Sara Galimberti, Virginia Ottaviano, Frederic Preffer, Mario Petrini

**Affiliations:** ^1^Division of Hematology, Department of Oncology, Transplants and Advances in Medicine, University of Pisa, 56126 Pisa, Italy; ^2^Department of Biochemistry and Medical Biotechnology, CEINGE Institute, Federico II University, 80145 Naples, Italy; ^3^Department of Pathology, Massachusetts General Hospital and Harvard Medical School, Boston, MA 02114, USA

## Abstract

Acute undiffentiated leukemia (AUL) is an acute leukemia with no more than one membrane marker of any given lineage. Blasts often express HLA-DR, CD34, and/or CD38 and may be positive for terminal deoxynucleotidyl transferase (TdT). The expression of CD34, HLA-DR, and CD38 has been shown in pro-T-ALL, although in this case, blasts should also express CD7 and cyCD3. However, some cases of T-ALL without CD3 in the cytoplasm and all TCR chain genes in germ line configuration are reported, features that fit well with a very early hematopoietic cell. We report a case of acute leukemia CD34+/−HLADR+CD7+CD38+cyCD3− in which a diagnosis of AUL was considered. However the blasts were also positive for CD99 and TCR delta gene rearrangement which was found on molecular studies. Therefore a differential diagnosis between AUL and an early cyCD3 negative T-ALL was debated.

## 1. Introduction

The distinction between Acute Undifferentiated Leukemia (AUL) and early Acute Lymphoblastic Leukemia (ALL) is complex and affects patient management options. AUL is an acute leukemia with no more than one membrane marker of any given lineage. It specifically lacks the T or myeloid lineage specific markers cyCD3 and MPO, respectively, and does not express B-lineage specific markers such as cyCD22, cyCD79a, or CD19. The tumor also lacks specific features of other hematopoietic lineages such as megakaryocytes or plasmacytoid dendritic cells. Blasts often express HLA-DR, CD34, and/or CD38 and may be positive for terminal deoxynucleotidyl transferase (TdT). The blasts are negative for myeloperoxidase and esterase [1]. The expression of CD34, HLA-DR, and CD38 has been shown in pro-T-ALL, although in this case, blasts should also express CD7 and cyCD3 (Table 1) [2]. However, some cases of T-ALL without CD3 in the cytoplasm and all TCR chain genes in germ line configuration are reported, features that fit well with a very early hematopoietic cell [3, 4]. We report a case of acute leukemia where a differential diagnosis between an early cyCD3-T-ALL and an AUL was debated.

## 2. Case Presentation

**Figure 1:**
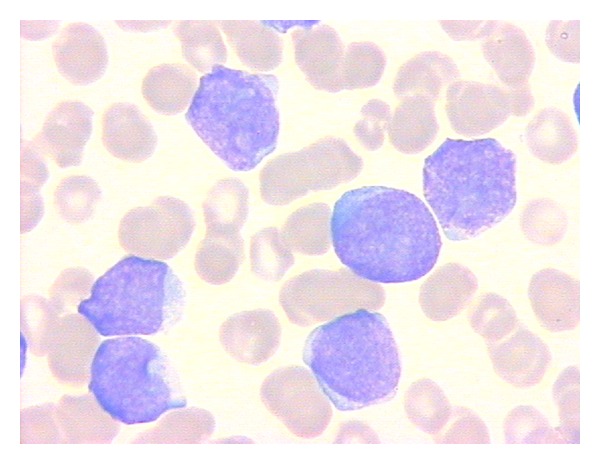
Blasts appear of medium large size, with nucleoli, without cytoplasmic granules.

**Figure 2:**
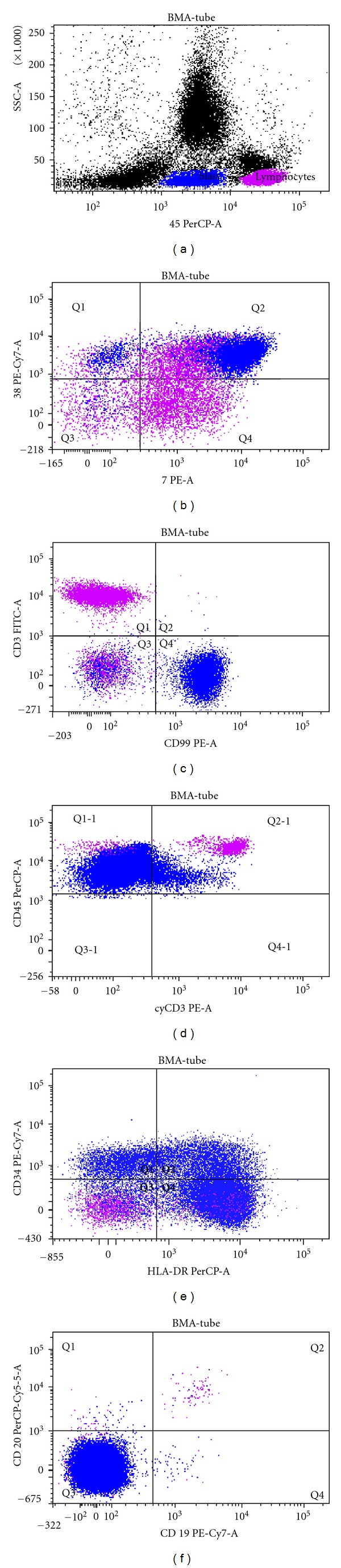
Blasts are shown in blue, lymphocytes in violet. Blasts are CD34+/−CD7+HLA−DR+CD38+CD99+CD3−cyCD3− CD19−CD20−.

A 65-year-old man was admitted to the hospital in November 2008 with a few weeks history of dyspnea and purpura with edema of the lower left limb. A complete blood count revealed: *White Blood Count *1,8 × 10^9^/l, *Hemoglobin* 9 g/dL, and *Platelet Count *10 × 10^9^/l. Manual differential showed 6% neutrophils, 66% lymphocytes, and 28% undifferentiated blasts with nucleoli and intensely basophilic cytoplasm. Splenomegaly and lymphadenopathy were present. Increased values of *lactic dehydrogenase (LDH)* (4.480 IU/l, n.v. 250–450) were registered. The bone marrow aspirate was hypercellular with blasts of medium-large size, with nucleoli, without cytoplasmic granules, and with occasional “hand mirror” forms (Figure 1). Cytochemical assays showed negativity for myeloperoxidase and *α*-naphthyl-acetate esterase and 20–30% of blasts to be PAS positive. Immunohistochemistry performed on the biopsy core showed that the tumor cells were CD79a-PAX5-CD68-MPO-CD3-CD7+CD34−. These results supported the diagnosis of leukocytosis of uncertain histologic origin. Flow cytometry was carried out by means of a FacsCanto II cytometer (Becton Dickinson) and samples were stained with fluorochrome-conjugated monoclonal antibodies (FITC, PE, PerCP, PE-Cy7, APC, and APC-Cy7) to the following antigens: CD3, cyCD3, CD2, CD7, CD1a, CD4, CD8, CD5, CD16-56, TCR*αβ*, TCR*γδ*, CD19, CD20, CD22, CD23, FMC7, CD10, CD38, CD45, *𝒦* and *λ* immunoglobulin light chains, cyCD22, cyCD79a, CD34, CD99, HLA-DR, CD117, CD33, CD13, MPO, CD45RA, and CD45RO. Data were collected and analyzed by FacsDiva software (Becton Dickinson). The blasts were positive for CD34+/−HLA-DR, CD7, CD99, CD38 (Figure 2), CD45RA, CD10+/−, and CD4 (not shown). Conventional cytogenetics performed on the bone marrow revealed a 47, XY karyotype with trisomy of chromosome 8 (47, XY, +8). T-cell receptor (TCR) delta gene rearrangements studies by polymerase chain reaction (PCR) showed the presence of a clonal TCR rearrangement and therefore a clonal T-cell population.

## 3. Discussion

The expression of CD34, HLA-DR, and CD7 has been shown in pro-T-ALL, although in this case, blasts should also express cyCD3 (Table 1) [2]. In AUL, blasts often express HLA-DR, CD34, and/or CD38 (1). In this case report the blasts were *CD34+/−HLA-DR+CD7+CD38+cyCD3− *such that a diagnosis of AUL was considered. However the blasts were also positive for CD99 and a TCR delta gene rearrangement, which was obtained from molecular studies. Thus, a differential diagnosis between an AUL and early cyCD3 negative T-ALL was considered.

Nishi et al. reported some cases of c-kit+ T-ALL without CD3 in the cytoplasm, and all TCR chain genes in germ line configuration, features that fit well with a very early hematopoietic cell [3]. Quintanilla-Martinez et al. also reported three cases of prethymic lymphoblastic lymphoma which resulted TdT+, HLA-DR+, CD34+, CD71+, CD38+, and CD7+, most resembling the normal prothymocyte. The prethymic T-cell character was further supported by germline T-cell receptor and chain genes [4]. In our case the blasts expressed CD99, which was reported as the most useful antigen to indicate the precursor nature of T lymphoblasts [5]. CD99 is intensely expressed in the earliest maturational stages of the myeloid and the lymphoid lineages. Dworzak et al. speculated that normal and leukemic cells could be distinguished in bone marrow and peripheral blood based on CD99, since maturation-related CD99 overexpression should be nonexistent or extremely rare on T cells outside the thymus [5]. Considering that normal bone marrow and/or early thymic cells are CD34+CD7+cyCD3− and are able to differentiate into myeloid and lymphoid cells [6, 7], the expression of CD7 and CD99 together on blasts negative for cyCD3 might be indicative of an earlier T-ALL than that of pro-T-ALL. *To support this hypothesis, Kawamoto et al. proposed a new model of hemopoiesis where, a common myeloid-lymphoid precursor was recognized *[8]. Cytogenetic and molecular studies could be helpful in conjunction with immunophenotype to confirm the T lineage of such leukemia. In this regard, our patient showed a TCR delta gene rearrangement. As reported in literature, CD7+ early T-cell acute lymphoblastic leukemia (T-ALL) shows a delta gene rearrangement, which occurs earlier than expression of cyCD3 epsilon protein products [9, 10]. Kimura et al. observed that DDJ delta rearrangements have never been observed in non-T-cell malignancies, such as precursor-B-ALL or acute myeloid leukemia [10]. Thus, TCR delta gene rearrangements studies represent a useful tool to establish lineage and clonality of leukemic cells in the most immature stages of T-cell development [10]. While anecdotal experience generally considers AUL to have a poor prognosis, information is too scanty to make any definitive statements about clinical features or outcome. T-ALL often presents with a large mediastinal mass or other tissue mass. Lymphadenopathy and hepatosplenomegaly are common. Our patient presented at diagnosis with lymphadenopathy and splenomegaly. Patients with ALL should have a lumbar puncture to exclude or confirm an involvement of the CNS with leukemia. All treatment protocols for ALL include a prophylaxis to the CNS by administrating drugs (e.g., methotrexate) to the intrathecal space [11]. Our patient did not have CNS involvement but achieved complete remission following an ALL type treatment, thus supporting our diagnostic hypothesis. In conclusion, the immunophenotypic study of a large number of T-ALL cases, in conjunction with associated clinical features, cytogenetic and molecular studies, would be helpful in differential diagnosis with AUL and might contribute to the choice of the most appropriate treatment and management for these patients.

## Figures and Tables

**Table 1:** T-ALL EGIL classification of T lineage acute lymphoblastic leukemia (T-ALL) modified by Szczepanski et al. [2].

MARKERS	Pro-T-ALL (EGIL T-I)	Immature thymocyte (EGIL T-II)	Common thymocytic T-ALL mCD3-(Cortical T-ALL, EGIL T-III)	Common thymocytic T-ALL mCD3+ (Cortical T-ALL, EGIL T-III)	Mature T-ALL (EGIL T-IV)
TdT	++	++	++	++	++
HLA-DR	+	−	−	−	−
CD34	+	−	−	−	−
CD1a	−	−	++	++	−
CD2	+	++	++	++	++
cyCD3	++	++	++	++	++
CD5	−	++	++	++	++
CD7	++	++	++	++	++
CD4−CD8−	++	+	−	−	−
CD4+CD8−	−	+/−	+/−	+/−	+
CD4−CD8+	−	+/−	+/−	+/−	+/−
CD4+CD8+	−	−	+	+	+/−
mCD3	−	−	−	++	++
TcR*αβ*	−	−	−	**60–70%**	**60–70%**
TcR*γδ*	−	−	−	**30–40%**	**30–40%**

<10% of the leukaemias are positive; +/−: 10–25% of the leukaemias are positive; +: 25–75% of the leukaemias are positive; ++: >75% of the leukaemias are positive.
